# A Comparative Study of the Effect of Peer-led and Lecture-based Education on Health Literacy in Patients with Multiple Sclerosis

**DOI:** 10.30476/IJCBNM.2020.85816.1298

**Published:** 2021-01

**Authors:** Ali Dehghani

**Affiliations:** 1 Department of Nursing, School of Nursing and Paramedical, Jahrom University of Medical Sciences, Jahrom, Iran

**Keywords:** Education, Health literacy, Lecture, Multiple sclerosis, Peer group

## Abstract

**Background:**

Patients with multiple sclerosis (MS) require health literacy to manage the symptoms and problems of the disease, which improves their quality of life. Health literacy is recognized as a critical indicator of health care outcomes. This study aimed to compare the effect of peer-led and lecture-based education on health literacy in MS patients.

**Methods:**

This quasi-experimental study was conducted on MS patients in Jahrom from December 2018 to November 2019. 90 patients were selected using convenience sampling and then assigned into control (n=45) and intervention groups (n=45). While routine education was presented to patients in the control group, peer education intervention was held for the intervention group. The number of sessions held for both groups was six sessions (one session per week). For data collection, MS health literacy questionnaire (MSHLQ) was used before and one month after the intervention in both groups. Data were analyzed through SPSS version 21 using Chi-square, Fisher’s exact test and t-test. The significance level was considered P<0.05.

**Results:**

Paired t-test showed that there was a significant difference between the mean of health literacy in the intervention group before and after the intervention (P=0.001), while this difference was not observed in the control group (P=0.39). Independent t-test showed a significant difference in the mean of health literacy between the intervention and control groups after the intervention (P=0.001).

**Conclusion:**

The results showed that peer group experiences were more effective than lecture-based education in improving health literacy. It can be beneficial to employ as an educative-supportive approach in MS patients.

## INTRODUCTION

Multiple Sclerosis (MS) is a chronic inflammatory disease of the central nervous system which affects sensory and motor functions. ^1^ MS is the most common neurological damage leading to disability in young adults, ^2 , 3^ and 80% of patients have some degrees of disability. ^4^ The number of patients with MS is about 2.5 million people worldwide in 2010, and 200 people are added weekly. ^4^ Iran has the highest prevalence of MS (51.52 per 100,000) in the Middle East and Asia. The prevalence of MS has increased significantly in Iran over the recent years. ^5 , 6^ According to the statistics of the MS Association in 2014, nearly 40,000 people suffer from MS in Iran. ^5^ The most common age affected by this disease is 20 to 40 years. ^7^

Prognosis of MS has remained unclear, and various physical and psychological problems that patients experience affect their daily activities, social and family life, functional independence, future planning, and feeling of wellness. ^8^ Chronic diseases such as MS affect the patients’ identity, psychosocial dimensions, emotional balance, self-satisfaction, sense of competence, and efficacy. It also affects social interactions which require increased information and health literacy in managing the symptoms and problems in order to improve the quality of life. ^8 - 10^ Consequently, health literacy has received attention over the recent decades. 

Health literacy is a global issue. According to the World Health Organization (WHO) statement, it plays a central role in determining health inequalities in both rich and poor countries. The WHO defines health literacy as “the cognitive and social skills which determine the motivation and ability of individuals to gain access to, understand and use information in ways which promote and maintain good health”. ^11^ The results of studies on patients with chronic diseases have shown that health literacy skills affect clinical outcomes such as anemia control, early management of metabolic and nutritional disorders, symptom management and complications, and delay in progression of chronic disease. Therefore, inadequate health literacy increases the health care inefficiency and inadequacy. ^12 - 14^ A study report showed health literacy as one of the most important opportunities for improvement of health. ^15^ Based on studies conducted by the Brooks et al., individuals with low health literacy do not understand the health care training and health recommendations; consequently, they have poorer health status and incur greater costs. ^16^ Therefore, increasing the level of health literacy in patients by an effective educational method is necessary to manage the symptoms and problems of the disease.

Most educational programs on MS have been presented individually in lectures, pamphlets, and booklets. Quality of these training programs is undesirable, and it is not responsive to clients due to the large number of visitors to MS societies and the small number of trained staff. ^17 , 18^ Group trainings have also been presented through lecture, which has consequences such as rapid forgetfulness, patient’s fatigue, and lack of opportunity for questioning and answering and motivation in patients, so that in this educational method, cooperation and intergroup relations are weak and individual differences are not taken into account. While today it is effective education that is associated with positive activities of the learner and leads to the acquisition of constructive experiences in the learner. ^18^ Therefore, it seems more appropriate to use a training program that can be provided in persons, which involves peer group. 

Peer-led education is defined as a tool employed by persons who share same experiences. ^19^ Peer education, as a method of patient education, has been confirmed effective in facilitating, advancing, and providing a place in which the patients receive their education. ^20^ Some studies have confirmed beneficial effects of peer education. ^19 , 21^ In a study conducted on 400 patients with chronic general medical conditions, it was indicated that employing peer education increases motivation and contributes to the reduction of psychological symptoms in patients. ^21^ Visiting the same patients with the same diagnosis would bring relief and assurance for patients to overcome the disease, leading to a higher life expectancy. ^22^ Most individuals recommend employing peer education because of its role in enforcing their identity and changing their views through role playing. ^19^ Peer-led education is one of the patient-centered and active teaching methods in which participants actively participate in educational activities using the discussion method and are given the opportunity to share their opinions and experiences with others. Teaching through peers increases the power criticism and learning in patients. ^23^ Therefore, given the importance of the subject and choosing the best educational method, this study aimed to compare the effect of peer-led and lecture-based education on health literacy in MS patients.

## MATERIALS AND METHODS

This is a quasi-experimental study with two non-randomized study groups, in MS Society Jahrom, South of Iran, from December 2018 to November 2019. Inclusion criteria were definite diagnosis of MS by a neurologist, willingness to participate in the study, age between 20 and 50 years (due to the distribution of the majority of MS patients in this age range), at least six months of living with MS, no history of dementia, confusion, mental and psychological problems which might hinder their participation, and patients with mild disability (Expanded Disability Status Scale (EDSS) between 0-1.5). The EDSS is based on the presence of certain symptoms in a typical neurological examination. These observations are evaluated on the scale from 0 to 5.5 in each functional system (FS) as the mild disability (0-1.5), moderate disability (2-3.5) and severe disability (4-5.5). The higher the EDSS score, the more profound the patient’s disability level. ^24^ The exclusion criteria were incomplete questionnaire, changes in the treatment process and drug used during the study, incomplete participation in peer and traditional training sessions, and participation in specific training program regarding managing the symptoms and problems of MS during the past 6 months. 

In this study, 90 patients were selected based on the inclusion criteria using convenience sampling from the MS Society and then assigned into control (n=45) and intervention groups (n=45). Considering the confidence level of 95% (Z1-α/2=1.96), power of 0.80, Mean±SD of 76.40±10.80 in the control group and Mean±SD of 82.60±8.90 in the intervention group, ^25^ we estimated the sample size in each group at 41 (the following formula). Assuming 10% of loss to follow-up, we decided to allocate 45 subjects in each group. 

$n=\frac{{\left(Z_{1-\frac{\alpha }{2}}+Z_{1-\beta }\right)}^{2}2\sigma^{2}}{{\left(\overset{-}{x_{1}}-\overset{-}{x_{2}}\right)}^{2}}=\frac{{\left(\mathrm{1.96}+\mathrm{0.86}\right)}^{2}\times 2\times \mathrm{97.91}}{{\left(\mathrm{82.60}-\mathrm{76.40}\right)}^{2}}=\mathrm{40.49}$

The data were collected using two questionnaires consisting of demographic and health literacy. The demographical questionnaire included age, gender, marital status, educational level, length of disease, relapse frequency during last year, type of MS, and type of drugs.

The specialized MS health literacy questionnaire (MSHLQ) was used to assess the health literacy before and one month after the last (sixth) training session in both groups. MSHLQ is merely specialized for MS and measures the health literacy of patients suffering from MS. The MSHLQ was developed by Dehghani and Keshavarzi in Persian in 2018.7 This scale consists of 22 items and four main dimensions including appraisal of health information, ability of health information search, knowledge of disease care, and successful functioning in health conditions. Each item was scored on a 5-point Likert scale, ranging from 0 (never) to 4 (always). The average health literacy score was between 0- 88. In general aspects of health literacy, higher scores shows better health literacy.

The validity of the MSHLQ was determined using the face, content, and construct validity method. Face validity of the scale was determined by asking 10 patients with MS about the level of difficulty, proportionality, and ambiguity of the items. The qualitative content validity about grammar, using appropriate words, placement of items in the appropriate place, and right scoring of the items were assessed by 15 experts. The content validity ratio (CVR) and content validity index (CVI) of the scale was determined by asking 15 experts about the necessity and relevance of each item, respectively. In total, the CVR and CVI of the questionnaire were 95.32% and 92⋅45%, respectively. Construct validity of the scale was also determined by factor analysis. Exploratory factor analysis using the principal component analysis (PCA) method and varimax rotation were used to determine the dimensions of the questionnaire. The Kaiser-Meyer-Olkin (KMO) test for the adequacy of sampling was obtained 0.932, which indicates adequacy of the sample. Bartlett’s test showed that there was a significant inter-item relationship at P=0.001. Therefore, by analysis of the structure validity and exploratory factor analysis, 22 items and 4 factors “appraisal of health information”, “ability to search the health information”, “knowledge of caring for the disease”, and “successful practices in health conditions” with eigenvalues above 1 were determined for the MS patient’s health literacy questionnaire. The four rotated factors explained 58% of the total variance. Reliability of the MSHLQ was also determined using internal consistency and stability method. Thirty patients with MS were invited to fill in the questionnaire and determine its reliability. The Cronbach’s alpha coefficient was 0⋅94. The stability of the scale using inter-class coefficient was 0⋅96. ^7^ The questionnaires were distributed and collected by the researcher.

Peers were selected from patients with MS who could participate in the study. The physician and psychiatrist of MS Society assisted in selecting the peer. Factors that were considered for selecting the peers were the following:

- Diploma and higher education
- Affliction with MS for at least five years
- A high level of health literacy using the health literacy questionnaire
- Appropriate social communication

According to the above-mentioned criteria, patients in the intervention group were divided into three 15-member groups. All three peer educators had a master’s degree and experience between 10-15 years of MS disease. The other characteristics is presented in Table 1.

**Table 1:** Characteristics of peer educators in the study

Characteristics	Age (years)	Gender	Education	Field of study	Experience of disease (years)	Job
Peer educator 1	48	Male	Master of science	Management	20	Employee
Peer educator 2	42	Male	Master of science	Educational science	17	Teacher
Peer educator 3	38	Female	Master of science	Psychology	14	Employee

The peers were educated using lectures and interactive discussions in three sessions. In the first session, concepts, importance, advantages of peer education, and communication skills were educated. In the second session, search of health information, access to health information, use of valid resources, and evaluation of health information were educated. In the third session, appropriate level of practice, control of symptoms, diagnosis, treatment and follow-up, complications of illness and medications, self-care, relaxation techniques, coping with the illness, diet, and social interactions were educated. In addition, the peers discussed the issues taught at the end of each session and their educational experiences.

As a therapeutically standard procedure, both intervention and control groups were given routine information in MS Society. Initially, all participants in both groups completed the health literacy questionnaires. In accordance with the results of the pre-test, educational content was designed and modified in cooperation with the experts. In the control group, the researcher (with MS degree in nursing) performed the training program during six sessions of 45-60 min that were held once a week in three 15-member groups. The training method in the control group was lecture, so that less information exchange and group discussion took place between the patients. In the intervention group, after the peers were prepared with three educational sessions, the three educated peers performed training program during six sessions of 45-60 min that were held once a week in three 15-member groups. Teaching methods in the intervention group included group discussion, question and answer, and interpersonal interactions. In the intervention group, the researcher only had a supervisory role during the sessions. The educational content was similar in both groups. Then, the patients of both groups were evaluated using the health literacy questionnaire one month after the intervention. Educational sessions in both groups were held in the MS Society. Topics of the educational intervention in each session is presented in Table 2.

**Table 2:** Topics of educational sessions in the two control and intervention groups

Session	Educational content
First session	Concept and nature of MS^a^
	Etiology and diagnosis of MS
Second session	Risk and recurrence symptoms during MS
	Treatment and therapies methods of MS
Third session	Side effects of MS drugs
	Management of medication side effects
Fourth session	Symptoms of MS
	Management of symptoms
	Relaxation techniques
Fifth session	Diet in MS
	Self - care in MS
Sixth session	Access and resource to health information
	Coping with MS

a Multiple sclerosis

Data were analyzed through SPSS version 21 software, using descriptive statistics including mean and standard deviation and inferential statistics including Chi-square tests, independent t-test, and paired t-test. The normality of the data was evaluated through two Sample Kolmogorov-Smirnov test. The significance level was considered 0.05.

The research was approved by the Research Ethics Committee at Jahrom University of Medical Sciences in Iran (Ethics Approval Number IR.Jums.Rec.1395.158). Before data collection, the participants signed a written informed consent. They were ensured about anonymity, confidentiality of the data, and voluntary withdrawal. It is necessary to mention that participation refusal in the study did not affect the process of receiving services.

## RESULTS

The results showed that patients in the intervention and control groups were similar in terms of age, marital status, educational level, etc. Mean and standard deviation of age were 33.62±8.03 and 31.64±7.10 in the control and intervention groups, respectively. Mean and standard deviation of the length of the disease were 8⋅15±5.50 and 6⋅45±5.50 in the control and intervention groups, respectively. Other information is presented in Table 3.

**Table 3:** Comparison of the patients’ demographic characteristics between the intervention and control groups

Characteristics		Groups		P value
		Intervention N (%)	Control N (%)	
Gender	Female	32 (71.10)	27 (60)	0.41*
	Male	13 (28.90)	18 (40)	
Marital status	Single	18 (40)	24 (53.30)	0.51**
	Married	27 (60)	21 (46.70)	
Educational level	Under diploma	9 (20.10)	4 (8.90)	0.91**
	Diploma	22 (48.80)	27 (60)	
	High diploma	14 (31.10)	14 (31.10)	
Relapse frequency during last year	Without relapse	8 (17.70)	9 (20.10)	0.15*
	Once	23 (51.10)	14 (31.10)	
	Twice	9 (20.10)	11 (24.40)	
	More than twice	5 (11.10)	11 (24.40)	
Type of MS^a^	Relapse-remitting	40 (88.80)	42 (93.30)	0.56*
	Secondary Progressive	5 (11.20)	3 (6.70)	
	Primary progressive	0 (0.00)	0 (00.00)	
Type of drugs	Moderator	27 (60)	24 (53.30)	0.45*
	Symptomatic	5 (11.10)	6 (13.40)	
	Synthetic	13 (28.90)	15 (33.30)	

* Chi-square test;

** Fisher’s exact test;

a Multiple sclerosis

Data have a normal distribution. Thus, the results of Kolmogorov-Smirnov test of health literacy for before and one month after the intervention in both group was P≥0.05. Comparison of the mean and standard deviation of health literacy before the intervention showed that there was no significant difference between the intervention and control groups (P=0.24). One month after the intervention, the patients’ health literacy was higher in the intervention group, compared with those in the control group (P=0.001) (Table 4). The effect size was 0.25 in the control group and 0.68 in the intervention group.

**Table 4:** Comparison of health literacy before and one month after the intervention between the intervention and control groups

Time	Before the intervention	One month after the intervention	P value*
Groups
Intervention	27. 50±8.75	67.10±5.33	0.001
Control	31.02±7.98	44.66±6.87	0.39
P value**	0.24	0.001	

* Paired sample t-test,

** Independent sample t-test

## DISCUSSION

In this study, an interactive patient education program as peer-led intervention was compared to a lecture-based education, information-only intervention for MS patients. As to the study outcome, health literacy, the peer-led interactive program proved to be superior to the lectures at the end of study. This finding is consistent with those of some studies. ^26 - 28^ The results of the study showed that both individual and peer-led educational methods led to promotion of the quality of life in patients with heart failure; however, the impact of peer educational method was stronger than individual education in long term. ^29^ The above studies show that training programs offered by peer groups can effectively encourage people to increase appropriate health behaviors. The results of another study showed that participation in peer-lead education program improved psychological aspects in people with chronic general medical conditions. ^21^ This finding was confirmed by other similar studies. ^30 - 34^ Consistency of these findings with the present study can be due to reasons such as the similarity of how to prepare patients as peer educators during 3 sessions. the number of training sessions in the intervention group, and presence of the researcher as an observer in peer training sessions. Also, findings could be due to the fact that peers belong to the same social group and people believe are similar in terms of ability, therefore can have a great impact on learning. The way of communicating with patients, as an acceptable and believable role model, and the simplicity of explaining for the educational contents are some of these effective reasons for changing the health literacy. Another reason for the findings of the present study could be different training methods in the two groups. For example, in the intervention group, there was more group discussions, question and answer, and interpersonal interactions, while only lecture was used as a training method in the control group. Therefore, the rate of learning in patient-centered methods such as peer-led education is higher than the lecture method, which is one of the advantages of learning in a patient-centered and active method compared with current educational patterns and one-way transfer of information. The results of the above studies in this field can suggest using peer groups instead of health care professionals to change the health behavior in patients, especially patients with chronic diseases such as MS. ^35^ The peer in our present study provided participants with emotional support and encouragement for daily management of their diseases. As a result, patients felt more confident in managing their disease. The reason for this improvement was reflected by the traditional and strong emotional bond between people in the Iranian culture. In Iran, individuals are committed to tradition, and people are expected to support one another. This traditional culture has highlighted the important role of peers in education. They use their experiences to provide other peers who are in trouble with an educational program and also, help the patients who do not have necessary skills to care for and control disease symptoms. The results of some studies were not consistent with those of the present study. ^36 - 38^ The results of a study showed that peer‐led education did not change the level of the quality of life and depression in patients with ankylosing spondylitis. However, because of the maintenance of the quality of life levels, this type of intervention may be considered as a supplementary intervention to the standard medical care for management of the disease. ^36^ The reason for the discrepancies in these findings could be due to the low sample size, the way of preparing the peer educator, and the low number of training sessions by the peers in the above study. Also, the results of another study showed peer- and nurse-led education methods did not yield much different results on stress of mothers of children with chronic diseases. Therefore, it is recommended that substitute peer-led training method should be changed into nurse-led training method, due to the nurses’ huge workload. ^37^ The reason for the discrepancies in these findings might be attributed to the fact that the researcher was present in the peerled group in the whole course of parent to parent training and monitored the accuracy of the exchanged information. The results of a study on MS patients showed that the total score of self-management and its dimensions increased significantly in both nurse-led and peer-led groups over time. The results also showed that over time, there was a significantly higher increase in the total self-management score, as well as health maintenance behaviors and following/avoiding the treatment scores in the nurse-led groups, compared to the peer-led groups. ^38^ One of the possible reasons for the ineffectiveness of peer-led education method in the above study may be related to the choice and training of the peer educator because one of the important criteria for the effectiveness of peer-led educational interventions goes back to the suitable peer educator who has sufficient ability and experience in teaching.

One of the strengths of the present study was its new approach to an educational method, which involved no complications for managing the disease and improved health literacy in MS patients. Also, as the other strength, it was found that low-cost and accessible teach-back training has potential benefits in providing more effective education compared with lecture education. The limitations of the present study were short-term follow-up after the intervention, personality characteristics, and culture of individuals in accepting educational content; also, we are not sure whether peer education occurred in the control group because peer education may occur at the end of the training sessions in the control group and there was more group discussion and interpersonal interactions in the intervention group.

## CONCLUSION

This study indicated that peer education could increase the health literacy among patients with MS. The results of the present study also showed that peer group experience compared with lecture-based educations had a significant effect on improvement of health literacy in patients with MS. It can be beneficial to be used as an educative-supportive method in MS patients. Therefore, the use of successful experiences of peer patients for education and improvement of another health dimension such as quality of life is recommended.
